# Outbreak report of SARS-CoV-2 infection by airborne transmission: Epidemiologic and molecular evidence

**DOI:** 10.7705/biomedica.6695

**Published:** 2023-03-30

**Authors:** María-Cristina Navas, Juan D. Cerón, Wbeimar Aguilar-Jiménez, María T. Rugeles, Francisco J. Díaz

**Affiliations:** 1 Grupo Gastrohepatología, Facultad de Medicina, Universidad de Antioquia, Medellín, Colombia Universidad de Antioquia Universidad de Antioquia Medellín Colombia; 2 Grupo Inmunovirología, Facultad de Medicina, Universidad de Antioquia, Medellín, Colombia Universidad de Antioquia Universidad de Antioquia Medellín Colombia

**Keywords:** SARS-CoV-2, COVID-19, disease outbreaks, vaccination, Colombia, SARS-CoV-2, COVID-19, brotes de enfermedades, vacunación, Colombia

## Abstract

**Introduction:**

It has been shown that the transmission of SARS-CoV-2 occurs mainly by air, and the risk of infection is greater in closed spaces.

**Objective:**

To describe the epidemiology, virology and molecular characterization of a COVID-19 outbreak at a closed vaccination point during the third wave of SARS-CoV-2 in Colombia.

**Materials and methods:**

Diagnostic tests, interviews, sampling, cell cultures and viral sequencing were carried out, the latter being molecular characterization and lineage identification.

**Results:**

Seven workers were positive for SARS-CoV-2; among these, 3 samples were analyzed, plus an additional sample belonging to the mother of the presumed index case; all samples were identified with lineage B.1.625, with a maximum of 2 nucleotides difference between them.

**Conclusions:**

Variant B.1.625 was identified as the cause of the COVID-19 outbreak, and a co-worker was also identified as the index case. Unexpectedly, attending a vaccination day became a risk factor for acquiring the infection.

## Resumen

**Introducción.:**

Se ha demostrado que la transmisión de SARS-CoV-2 se produce principalmente por vía aérea y el riesgo de infección es mayor en espacios cerrados con alta concentración de personas; este último factor se presentó en algunos de los puestos de vacunación de la ciudad de Medellín.

**Objetivo.:**

Describir la epidemiología, virología y caracterización molecular de un brote de COVID-19 en un punto de vacunación cerrado durante la tercera ola de SARS-CoV-2 en Colombia.

**Materiales y métodos.:**

Se realizaron test diagnósticos, entrevistas, toma de muestras, aislamiento viral y secuenciación genómica. Con esta última, se hizo la caracterización molecular y se identificó el linaje.

**Resultados.:**

Siete trabajadores fueron positivos para SARS-CoV-2, y de estos, tres muestras fueron secuenciadas, más una muestra adicional perteneciente a la madre del presunto caso índice. Todas las muestras fueron identificadas con el linaje B.1.625, con un máximo de dos nucleótidos de diferencia entre ellas.

**Conclusiones.:**

Se identificó la variante B.1.625 como la causante del brote de COVID-19, y también un compañero de trabajo fue identificado como el caso índice. De forma imprevista, asistir a una jornada de vacunación se convirtió en un factor de riesgo para adquirir la infección.

In Colombia, the first Coronavirus Infectious Disease 19 (COVID-19) case was detected and confirmed on March, 2020, on a person traveling from Italy. The country has since then experienced several waves of SARS-CoV-2 infections. Since the beginning of the pandemic, more than five million cases and 129,487 fatal cases had been reported by the Colombian *Ministerio de Salud y Protección Social* by the end of 2021 ^1^^,^^2^. The third wave that occurred from April to July, 2021, was the more severe in Colombia, with the highest incidence and deaths reported up to date ^3^.

The analysis of the genomic surveillance during the third wave demonstrated that the variant of interest (VOI) mu was the country’s most important lineage of the SARS-CoV-2 ^4^. Indeed, the Colombian *Instituto Nacional de Salud* characterized the emergence of the B.1.621 lineage in January, 2021, which was identified as the VOI Mu by the World Health Organization (WHO). Furthermore, the characterization has shown that mutations in the spike protein, such as E484K, N501Y, and P681H, could be related to the disease burden and its epidemiological impact ^3^.

The pandemic mostly affected the low-to-middle-income population because of its economic impact. Since the country has had long periods of lockdowns and travel restrictions, the government needed to reopen many restricted activities. Overall, the economic reopening, the progressive erosion of biosafety and insulation standards and the emergence of the mu variant could have been the most important factors related to the strength of the third wave in the country.

During the third wave, a group of co-workers presented signs and symptoms associated with SARS-CoV-2 infection. They had previously been appointed at the same time for the first dose of vaccine against COVID-19 in Medellín (the capital of the department of Antioquia, Colombia). The facilities and conditions of the vaccination room did not allow strict compliance with the regulations of distancing, ventilation and waiting time.

These conditions, added to the evidence of the transmission of SARS- CoV-2 mainly by aerosols and small droplets during close contacts, and the time between the vaccination and the onset of symptoms in the group of workers, makes us to suspect that they acquired the infection in the vaccination room due to the aforementioned conditions ^5^^,^^6^. A study was designed with the aim of characterizing epidemiologically, virologically and molecularly this outbreak of COVID-19.

## Materials and methods

This was a retrospective, descriptive, transversal study.

### Sample collection and analysis

We interviewed 24 co-workers who attended the appointment for their first vaccination dose. Demographics and clinical data from the people involved in the study were obtained through social networks and virtual meetings with the workmates.

After the vaccination and the suspected contagion, 13 out of 24 coworkers underwent COVID-19 diagnostic testing (antigen and/or RT-qPCR) in different health institutions in Medellín (table 1). Furthermore, nasopharyngeal swabs samples were obtained from four co-workers and a family member of the index case, five to eleven days after the vaccination, for additional SARS- CoV-2 studies, including further antigen/RT-qPCR testing, viral isolation and sequencing. Informed consent was obtained from each participant.

**Table 1:** Epidemiological, clinical and laboratory data of co-workers attending the indoor vaccination appointment

Person	Mask type and other protection	Age/Sex	Antigen test	qPCR	Date of onset of symptoms	Symptoms and Evolution
# 5	One layer cloth mask	49/Female	ND*	Negative	NA**	No symptoms
# 6	Three layer cloth mask	63/Female	ND	Positive	12-04-21	Fever, cough, chills, sweating, myalgias, fatigue, dyspnea, pneumonia
# 10	One layer cloth mask and safety glasses	ND	Negative	ND	NA	No symptoms
# 12	N95	54/Male	ND	Positive	10-04-21	Fatigue, headache, rhinorrhea, dysesthesias in legs and scapular area
# 13	Three layered cloth mask	56/Female	Positive	ND	11-04-21	Fever, cough, chills, fatigue, headache, anosmia, ageusia, diarrhea
# 14	Surgical mask and safety glasses	56/Female	ND	Positive	12-04-21	Fatigue, headache, back pain, cough, anosmia, ageusia
# 15	cloth mask	48/Female	Positive	ND	09-04-21	Headache, fatigue, fever, cough, anosmia
# 16	Three layer cloth mask	42/Female	Positive	ND	16-04-21	Fever, cough, chills, fatigue, headache
# 17	N95 and safety glasses	ND	ND	Negative	NA	No symptoms
# 18	Surgical mask and safety glasses	ND	ND	Negative	NA	No symptoms
# 20	Surgical mask	ND	ND	Negative	NA	No symptoms
# 23	Surgical mask	63/Male	ND	Positive	12-04-21	Fever, cough, chills, fatigue, headache, respiratory distress, severe bacterial pneumonia
# 24	N95 and surgical mask	ND	ND	Negative	NA	No symptoms

* ND: Not done/no data

** NA: Not applicable

For the qualitative detection of SARS-CoV-2, samples were tested using a rapid immunochromatographic assay for the viral nucleocapsid antigen, standard Q Covid-19 Ag test (SD Biosensor).

For the molecular detection viral RNA extraction was performed from samples using the QIAamp Viral RNA Mini Kit™ (Qiagen, Hilden, Germany). For molecular detection, the RNA extracts were amplified using qScript XLT 1-Step RT-qPCR Tough Mix™ (Quantabio Beverly, MA, USA) and the SARS- CoV-2 N1 primers of the US Centers for Disease Control (CDC) RT-PCR protocol (IDT, Coralville, lW, USA ^7^.

For viral isolation, Vero-E6 cells were seeded 24 hours before and a fraction of the nasopharyngeal samples (100 *μ*l diluted in 250 *μ*l DMEM) were inoculated over 75% confluent monolayers. Inoculum and cells were incubated at 37° C in 5% CO_2_ atmosphere for 90 minutes with gentle shaking every 30 minutes. The inoculum was removed and replaced with 1,5 ml DMEM medium containing 2% FBS and 1% penicillin-streptomycin. Cultures were monitored daily under the microscope to visualize the cytopathic effect ^8^*.* Viral RNA was extracted from supernatants, and the presence of SARS- CoV-2 on cultures was confirmed using qRT-PCR as explained above ^9^. This process was carried out to increase the number of viral particles per sample, improving the completeness of sequencing.

For the genomic characterization of the virus, next-generation sequencing was performed following the ARTIC protocol ^10^ at Corpogen Laboratories, Bogotá, Colombia. Briefly, amplicons were quantified and labeled with the Native Barcoding Kit EXPNBD104™ (Oxford Nanopore Technologies, Oxford, UK) and pooled in an equimolar amount. Genomic libraries were prepared with the SQK-LSK109™ 1D ligation kit (Oxford Nanopore Technologies) and sequenced using a FLO-MIN106-R9.4™ flow cell and the MinION™ instrument (Oxford Nanopore Technologies). Bases were identified using Guppy, version 3.2.2 ™ (Oxford Nanopore Technologies). The processed reads were aligned with the SARS-CoV-2 reference genome (GenBank NC_045512.2) using the BWA-MEM 14 algorithm and the BBMap ^11^ to generate the consensus sequence. The viral lineage was classified using PANGOLIN (Phylogenetic Assignment of Named Global Outbreak LINeages) ^12^.

For the phylogenetic analysis, the complete sequences associated with the outbreak and other 19 sequences from unrelated COVID-19 patients from Medellín were aligned with reference sequences of the original Wuhan 2019 isolate, variants of concern (VOC) and VOIs. Sequences identified as lineages B.1.111 and B.1.625 from the GISAID database [https://www.gisaid.org/ ] were also included because these lineages were highly prevalent in Medellín during 2021 ^4^.

A total of 204 sequences were analyzed (dataset available from the authors upon request). The alignment by the MUSCLE algorithm and the selection of the substitution model by the Bayesian information criterion were carried out with MEGA 11 software ^13^. Phylogenetic analysis was performed by the maximum likelihood method on the IQ-TREE web server ^14^ with the GTR + G + I substitution model and an ultrafast bootstrap of 5,000 replicates. The resulting tree was visualized on the iTOL website ^15^.

This study was designed and conducted according to the Declaration of Helsinki and the Colombian legislation (Colombian *Ministerio de Salud Resolution* 008430, 1993). All subjects signed an informed consent. The collected biological material and demographic data were codified to ensure the privacy of the study participants.

### Statistical analyses

The secondary attack rate was estimated as the number of COVID-19 cases diagnosed divided by the total number of people at risk, and 95% confidence intervals were calculated using the binomial exact method.

## Results

**Figure 1:**
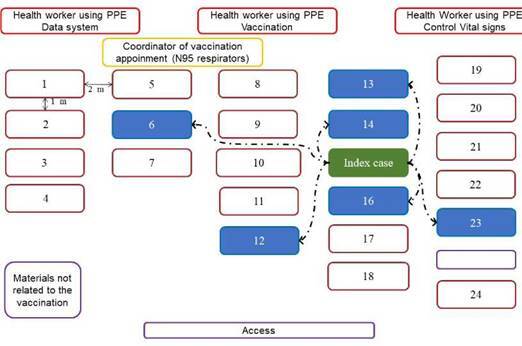
Schematic diagram of vaccination appointment location. The distribution of the study population during the vaccination appointment, the distance between rows and chairs, and the location of the index and source person are presented in this figure. The rectangles correspond to each one of the people present in the room: health personnel highlighted in red, coordinator of vaccination appointment in yellow, index case in green, co-workers who were infected in this outbreak in blue and other co-workers in brown. The double arrows indicate the distance between each attendee and the dotted arrows indicate the contagion link between index case and those affected.

The vaccination appointment of the co-workers was scheduled for April 8, 2021, in a meeting room enabled for that purpose. Due to the logistics and requirements of vaccination, the workers stayed about an hour in the room, which had an area of 105 m^2^. That room did not have air conditioning, and its ventilation was limited to a system of windows and lattices. The distribution of the workers and the health personnel, the distance between rows and chairs, and the location of the index case are presented in figure 1. Everyone in the room wore a mask, although of different type and quality, such as cloth masks (one, two or three layers), surgical masks or N95 (table 1). There was an active conversation among most of the attendees, but physical contact was avoided.

Seven out of 24 co-workers attending the SARS-CoV-2 vaccination session developed COVID-19 clinical symptoms, four to ten days after the exposure. Symptoms included headaches, fever, chills, myalgia, cough and anosmia. Clinical evolution and virological data of the seven cases were variable, but symptomatology related to COVID-19 lasted around 14 days in most cases. Two cases presented with pneumonia, and one required hospitalization (table 1). The diagnosis was confirmed by antigen or viral genome detection in all cases; indeed, the secondary attack rate was 25% (95%CI: 9.8-46.7%). Subsequently, COVID-19 cases, one of them fatal, occurred in five relatives who had contact with two cases of the outbreak. The other six co-workers who underwent SARS-CoV-2 testing were negative for viral genome detection by RT-qPCR, and none of them presented symptoms (table 1).

**Figure 2:**
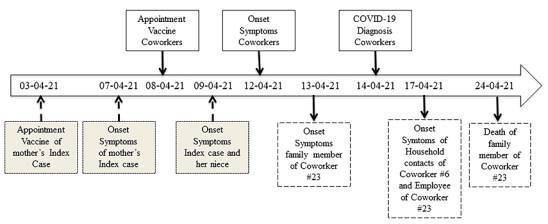
Contagion timeline of the indoor vaccination appointment outbreak in Medellín, Colombia. The timeline describes the most important events associated with the viral outbreak, the data was collected through interviews as described in the materials and methods section.

The index case, and presumed source of this outbreak, was a female that was asymptomatic at the vaccination date but presented headache the next day; her mother had developed headache the day before the co-workers’ vaccination but it was assumed as a side effect of the COVID-19 vaccination that she received 96 hours before. Moreover, a niece of the index case also developed symptoms. The diagnosis of COVID-19 was laboratory confirmed in the index case and her family members 7 to 11 days after the mother’s vaccination (figure 2).

Positive viral isolation for SARS-CoV-2 was obtained from four of the workmates and additionaly from the mother of the index case. These isolates were subjected to whole genome sequencing. All genome sequences were preliminarily identified as lineage B.1.625 on the Nextclade web server tool ^16^. One of these genomes (case # 6) had low coverage (<50%) and was not included in the final analysis. The remaining four viral genome sequences from cases #13, #16, #23 and from the mother of the index case, are in GISAID database with access codes EPI_ISL_13902608, EPI_ ISL_13902607, EPI_ISL_13902609 and EPI_ISL_13902610, respectively. All had coverage greater than 81%; three of them were identical to each other, and the fourth (case #16) differed in only two positions.

**Figure 3:**
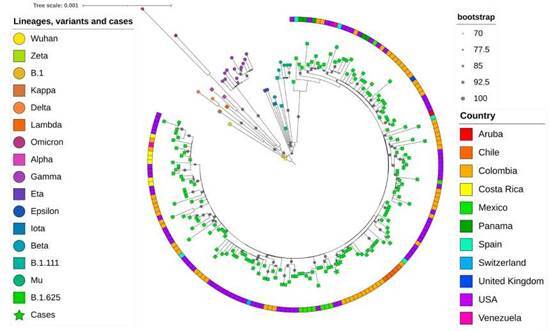
Phylogenetic tree of the sequences obtained in this study and other reference sequences. The different variants, lineages and cases were colored according to the key in the left panel. The four sequences associated with the outbreak are indicated as green stars. The sequence B.1 corresponds to the first isolation of SARS-CoV-2 in Colombia ^8^. The size of the gray circles indicates the strength of the branch support according to the upper right panel. The boxes on the outside of the circle indicate the country of origin according to the colors in the lower right panel. The analysis was performed by the maximum likelihood method in the IQ-Tree server.

In the inferred phylogenetic tree (figure 3), a clear differentiation of the lineage B.1.625 was observed; the four genomes associated with the outbreak formed a monophyletic group within the B.1.625 clade with bootstrap support of 100. These sequences showed the characteristic spike mutations of this lineage, including del69-70, T95I, del144, F157L, N440K, E484K, D614G, D950N and V1228L. Other sequences were obtained from patients attending health institutions in Medellín, but not associated with the outbreak, grouped with sequences of variants alpha, gamma, mu, delta, B.1.111 or B.1.625 in the phylogenetic analysis.

## Discussion

We described the epidemiology, virology and molecular characteristics of an indoor SARS-CoV-2 outbreak. We identified seven cases of COVID-19 among the 24 co-workers attending a vaccination session, corresponding to a secondary attack rate of 25% (95% CI: 9.8-46.7%). The diagnosis of COVID-19 was also confirmed in five household contacts of two co-workers and two family members (mother and niece) of the index case. According to the contagion timeline, 13 cases were related to the mother of the index case. In turn, this 75-year-old woman may also have been infected during her vaccination that occurred in an overcrowded place five days before the vaccination of co-workers. Still, the details of this contagion were communicated informally and not thoroughly investigated (figure 2).

The logistics and biosecurity conditions of the vaccination sessions of the workmates were not appropriate to prevent the transmission of SARS-CoV-2, a respiratory virus usually transmitted by large respiratory droplets and aerosols. First of all, the vaccination session of co-workers was performed in a room with biosafety limitations such as suboptimal ventilation; the distance between rows was around two meters, but the distance between the chairs of the same row was only around one meter (figure 1). Moreover, the number of persons at the appointed place, the time spent in the room (30 to 90 minutes) and the active conversation among some attendees during the appointment, including the index case, could have facilitated the airborne transmission.

All vaccination attendees wore face masks at all times, however these were uneven in quality, ranging from single-layer cloth mask to N95s (table 1). The low number of exposed and infected individuals does not allow to draw conclusions about the relative efficacy of the various types of mask but it was notorious that infections occurred in people wearing all kinds of masks. Notwithstanding the foregoing, there is ample evidence that the N95 provides the best protection.

Although droplet and fomite transmission of SARS-CoV-2 were considered the most frequent modes for contagion at the beginning of the pandemic, several studies support the relevance of SARS-CoV-2 aerosol transmission. Evidence include super-spreading events, virions detection on the air, differences between outdoor and indoor transmission, detection of the virus in filters and ducts, cases in health workers despite using personal protection equipment that prevent droplet but not aerosol exposure and animal infection located in separately cages, among others ^6^. Indeed, virions of SARS- CoV-2 have been detected in aerosols ^17^^-^^20^, and a half-life of one to three hours of the virions in aerosols was estimated ^21^. Moreover, WHO and the US Centers for Disease Control and Prevention (CDC) recognize the airborne transmission of SARS-CoV-2 at short and long ranges ^22^^,^^23^.

Aerosols are particles (liquid, solid or semisolid) less than 5 pm in size that could stand suspended in the air for a while. These particles are normally generated during breathing, talking, laughing, singing, shouting, sneezing and coughing from individuals with or without respiratory infections. Furthermore, the conditions related to virus transmission by aerosols include the viral load, stability of the virions, size distribution of particles, electric charge, air/liquid interfacial properties and the composition of aerosols such as electrolytes, proteins and surfactants ^6^.

Variables to be considered for the route transmission by aerosol inhalation in this outbreak are the average temperature (21.5 ^o^C) and relative humidity (63-73%) of Medellin ^24^, the indoor environment with suboptimal ventilation, the number of people per square meter, the time spent at the room, the distance between chairs and rows, and the active conversation among persons. Although other routes of transmission, such as transmission by direct contact or by fomites, cannot be ruled out, their probability would be very low since direct contact was limited and objects were not shared during the appointment.

The high nucleotide identity and the monophyletic grouping of the sequences of the three co-workers (cases # 16, # 13 and # 23) and the mother of the index case support a common source of contagion in this outbreak. The phylogenetic analysis of the viral sequences demonstrated the B.1.625 lineage of SARS-CoV-2. The Colombian *Instituto Nacional de Salud* described this variant for the first time in January, 2021 (https://github.com/cov-lineages/pango-designation/issues/93 ). Afterward, this variant was reported in countries such as Venezuela, Dominican Republic, Mexico, Aruba, USA, Germany, Spain and England (GISAID sequences up to 2021-05-25) ^25^.

In addition to the D614G mutation at the virion spike, exhibited in most circulating variants, and associated with greater infectivity, other spike substitutions and deletions (del) present in the sequences of this outbreak have been associated with some phenotypic trait: del 69-70, located at the N-terminal domain (NTD) is associated with greater transmissibility. Del 144, located at an NTD epitope formed by amino acids 140-156, is potentially related to immune escape. Two other observed mutations, N440K and E484K, are located at the receptor binding domain and potentially affect the affinity or the antigenicity of the virus; in particular, E484K has been associated with immune escape and increased affinity to the human ACE2 receptor ^26^. The substitution E484K has also been characterized in VOCs such as beta and gamma ^27^^,^^28^.

The Colombian genomic surveillance network for SARS-CoV-2 led by the *Instituto Nacional de Salud* reinforced the monitoring since January, 2021, because of the high probability of importation of circulating VOCs and VOIs. The approach was sequencing and genomic characterization of a probabilistic sample of COVID-19 cases from all regions of the country. The first sampling batch was performed between April 15^th^ to June 15^th^, 2021, which coincided with the third and most severe wave of SARS-CoV-2 infection in Colombia.

A total of 1,630 samples from 27 regions/departments of Colombia were processed. Finally, 1,101 sequences were available in GISAID database. The phylogenetic analysis demonstrated the predominance of mu VOI (52,7%), followed by the gamma VOC (23.3%), B.1.625 variant (8.6%), and alpha VOC (5.7%).

**Figure 4:**
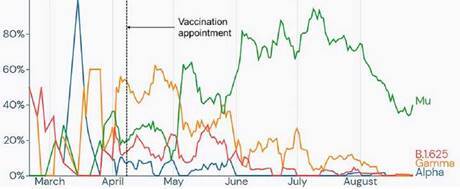
Frequency of the reported genomes of the alpha, gamma and mu variants and the B.1.625 lineage in Antioquia from February to September 2021. Frequency is based on genomes reported on the GISAID platform, the time frame corresponds to the first and last reported date of lineage B.1.625, and the frequencies of alpha, gamma and mu variants were added as they are the most reported during the time frame. The dotted line indicates the date of the outbreak.

Source: https://outbreak.info/.

This result indicated that variant B.1.625, causing the outbreak described in the present study, was a minority but still a circulating variant during the third epidemic wave in Antioquia. Interestingly, variant B.1.625 disappeared in the countrywide sampling carried out from September 15^th^ to October 30^th^, 2021 (figure 4).

In summary, we provide epidemiological and molecular evidence of an outbreak of infection by SARS-CoV-2 variant B.1.625 acquired during an indoor vaccination session. Although we emphasize that the contagion occurred in an activity that, paradoxically, was intended to prevent the disease, we are not suggesting that vaccination is a risky practice by itself. Vaccination has been shown to be highly effective in reducing severity and mortality, as reported in the technical report number 3 “COHORTE ESPERANZA” presented by the Colombian *Ministerio de Salud y Protección Social*. Vaccination is a safe practice provided that appropriate biosafety measures are strictly followed during epidemic situations.
